# Evaluation and Screening of Hypoglycemic Activity of Total Ginsenosides GBE-5 Fraction From *Panax Ginseng* Berry Based on UHPLC–MS Metabolomics

**DOI:** 10.3389/fnut.2022.865077

**Published:** 2022-04-25

**Authors:** Heyu Wang, Yu Tong, Anqi Wang, Ying Li, Bofan Lu, Hui Li, Lili Jiao, Wei Wu

**Affiliations:** ^1^Jilin Ginseng Academy, Changchun University of Chinese Medicine, Changchun, China; ^2^School of Pharmacy, Jilin Medical University, Jilin, China

**Keywords:** ginseng berry, ginsenoside, UHPLC-MS, metabolomics, type 2 diabetes mellitus

## Abstract

**Objective:**

Ginseng berry (GB) was the mature fruit of medicinal and edible herb, *Panax ginseng* C.A. Meyer, with significant hypoglycemic effect. Ginsenoside was the main hypoglycemic active component of GB. Evaluating and screening the effective components of GB was of great significance to further develop its hypoglycemic effect.

**Methods:**

The polar fractions of ginseng berry extract (GBE) were separated by a solvent extraction, and identified by ultra-high performance liquid chromatography–high-resolution mass spectrometry (UHPLC–MS). The insulin resistance model of HepG2 cells was established, and the hypoglycemic active fraction in GBE polar fractions were screened *in vitro*. Rat model of type 2 diabetes mellitus (T2DM) was established to verify the hypoglycemic effect of the GBE active fraction. The metabolomic study based on UHPLC–MS was used to analyze the differential metabolites in the serum of T2DM rats after 30 days of intervention with hypoglycemic active GBE fraction. The kyoto encyclopedia of genes and genomes (KEGG) metabolic pathway enrichment analysis was used to study the main metabolic pathways involved in the regulation of hypoglycemic active parts of GBE.

**Results:**

It was found that GBE-5 fraction had better hypoglycemic activity than other GBE polar fractions *in vitro* cell hypoglycemic activity screening experiment. After 30 days of treatment, the fasting blood glucose value of T2DM rats decreased significantly by 34.75%, indicating that it had significant hypoglycemic effect. Eighteen differential metabolites enriched in KEGG metabolic pathway were screened and identified in the rat serum from T2DM *vs*. GBE-5 group, and the metabolic pathways mainly involved in regulation include arachidonic acid (AA) metabolism, linoleic acid (LA) metabolism, unsaturated fatty acid biosynthesis, and ferroptosis.

**Conclusions:**

The hypoglycemic effect of GBE-5 fraction was better than that of total ginsenoside of GB. The AA metabolism, LA metabolism, unsaturated fatty acid biosynthesis, and ferroptosis were the potential metabolic pathways for GBE-5 fraction to exert hypoglycemic regulation.

## Graphical Abstract

**The main experimental process of this study**. 
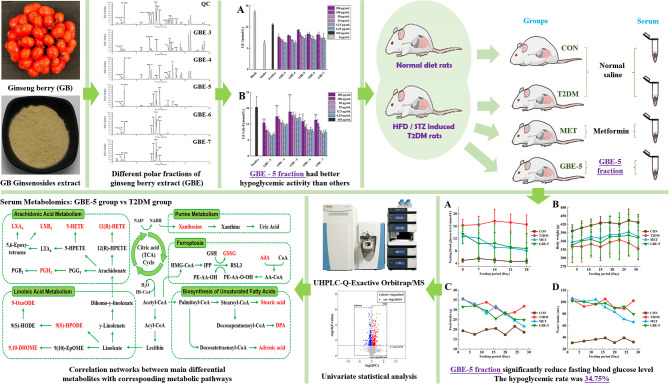

## Introduction

Ginseng (*Panax ginseng* C.A. Meyer), a perennial herb in the genus *Panax* of the Araliaceae family, has been used as herbal medicine, functional food, or dietary supplement in Asian countries for thousands of years, which was the globally top-selling herbal product (1). As a natural plant-based supplement, ginseng has gradually developed many ginseng-based foods, such as ginseng beverage, ginseng tea, ginseng sugar, and ginseng honey tablets (2). Ginseng berry (GB) was the mature fruit of *P. ginseng*, which could be harvested from the year of fourth of the ginseng planting (3). The main active component of GB were ginsenosides, which had anti-cancer, anti-oxidant, anti-inflammatory, immune modulation, hypoglycemic, and anti-diabetic effects (4–10). The content of ginsenosides in GB was more abundant than that in ginseng root (3); therefore, it has shown a stronger effect in anti-diabetic (2), which has been developed as a clinical drug for treatment and improvement of type 2 diabetes mellitus (T2DM) and its complications, such as Zhenyuan capsule, the main ingredient was GB total ginsenosides extract, had good hypoglycemic and hypolipidemic effects (11).

The research showed that the content of ginsenoside in GB was 4–10 times that of ginseng root (12), and the content of ginsenoside Re was 7–30 times that of ginseng root (13). It was reported that the content of ginsenoside Re in GB accounts for 85% of the total ginsenosides (11), which meant that ginsenoside Re may be the main active component of GB. In previous studies, the hypoglycemic activity of total ginsenosides extract of GB was verified (7), it was found that GB could significantly reduce the fasting blood glucose in T2DM rats, and the hypoglycemic rate was 27.35%. Further research on ginsenoside Re found that although Re was the main component of GB, its hypoglycemic rate was lower than GB, which was 21.97% (14), indicated that the hypoglycemic effect of GBE was not caused by a single component, but by multiple active components acting on multiple targets so as to play the role of reducing blood glucose. In general, the latest research on the hypoglycemic effects of ginseng and American ginseng mainly focused on total saponins (7, 15) or single ginsenoside (14, 16, 17), while there were few studies on hypoglycemic combination components. Among the reported hypoglycemic mechanisms of ginseng, relevant studies mainly focused on the level of cell signal transduction pathway, while there were few studies based on the mechanism of metabolic pathway. Therefore, it was necessary to screen the pharmacodynamic material basis of hypoglycemic effect in GB in order to further develop and utilize its excellent hypoglycemic activity.

Type 2 diabetes mellitus was mainly caused by insulin resistance or insufficient insulin secretion (18), ginsenosides could promote insulin secretion, increased insulin sensitivity, and reduced blood glucose concentration, but will not increase the risk of hypoglycemia (19). In the study of diabetes, the T2DM rats induced by high-fat and high-sucrose (HFD) diet combined with streptozotocin (STZ) was usually used as experimental animal models *in vivo*, and the STZ destroyed rat islet β-cell to simulate the state of insufficient insulin secretion (20, 21). It was stated that HepG2 was a popular hepatic cell line, its surface contained high-affinity insulin receptor, which could meet the requirements of typical insulin receptor (22), and *in vitro*, the HepG2 human hepatoma cells were usually used to establish insulin resistance model to simulate insulin resistance in the patients with diabetics. There were three main modeling methods of HepG2 cell insulin resistance model, high-glucose induced cell insulin resistance model, high-concentration insulin induced cell insulin resistance model, and fatty acid (palmitic acid) induced cell insulin resistance model (23). In recent years, scholars often use fatty acid method for relevant experimental research (24), and compared with other modeling methods, it has more reliable characteristics.

In this study, cell model activity screening combined with metabolomics was used to explore the active components of GB total ginsenosides, which had good hypoglycemic activity. As shown in Graphical Abstract, the polar segmentation of the total ginsenosides GBE was separated by organic solvent extraction, and the insulin resistance model of HepG2 cells was established, and the hypoglycemic active fraction of GBE was screened *in vitro*, then the hypoglycemic activity was verified by T2DM rat model. The univariate statistical analysis based on UHPLC–MS was used to screen the differential metabolites in rat serum after 30 days of intervention. Combined with the enrichment analysis of KEGG metabolic pathway, the potential targets of hypoglycemic active fraction of GBE were explored.

## Materials and Methods

### Material and Reagents

The *P. ginseng* berry was purchased from Ji-An (Jilin, China) in June 2019, the botanical identification was undertaken by Professor L. Jiao, and the voucher specimen (No. 20190002) was kept at the Jilin Ginseng Academy, Changchun University of Chinese Medicine, China. Ginseng berry total ginsenosides extract was prepared according to the literature method (7). Also, HepG2 human hepatoma cell line was purchased from Shanghai Institute of Biochemistry and Cell Biology, Chinese Academy of Sciences (Shanghai, China).

Petroleum ether, ethyl ether, ethyl acetate–ethyl ether and *n*-butanol were purchased from Beijing chemical works (AR, Beijing, China). Sodium hydroxide (NaOH) was purchased from Tianjin Xintong Chemical Co., Ltd (AR, Tianjin, China). Streptozotocin was purchased from Sigma chemical Co. (St. Louis, MO, USA). Citric acid and sodium citrate were purchased from Beijing Taibo Chemical Co., Ltd. (Beijing, China). Noto-ginsenoside R_1_, Ginsenoside Rg_1_, Re, Rf, Rb_1_, Rc, Rb_2_, Rb_3_, Rd, F_2_, Rg_3_ (purity ≥ 98%) were purchased from the National Institutes for Food and Drug Control (Beijing, China). The UHPLC-grade acetonitrile and formic acid were purchased from Fisher Scientific (Waltham, MA, USA). Ultrapure water was filtered using a Milli-Q device (Millipore, Milford, MA, USA). Cell Counting Kit-8 (CCK-8) was purchased from Dojindo Chemical Co. (Dojindo, Kumamoto, Japan). Bovine serum albumin (BSA) was purchased from Shanghai Yuan-ye Bio-Technology Co., Ltd (Shanghai, China). Glucose assay kit were obtained from Nanjing Jiancheng Biotech. Co., Ltd. (Nanjing, China).

### Preparation of Different Polar Fractions of Ginseng Berry Extract

Weighed 30 g of total ginsenoside from ginseng berry extract (GBE), added 300 ml of distilled water and dissolved in 60°C water bath, placed it in 1,000 ml separatory funnel, and added different organic reagents to extract it in turn. First, petroleum ether was added and extracted for 3 times, 300 ml each time, after combination, ginseng berry extraction I (GBE-1) was obtained. Added ethyl ether solution, extracted 3 times, with the volume of 300 ml each time, and combined to obtain ginseng berry extraction II (GBE-2). Added ethyl acetate–ethyl ether (3:2, v/v) solvent and extracted for 3 times. The addition volumes were 300, 200, and 150 ml, respectively. The extraction of ginseng berry extraction III (GBE-3) was obtained by merging the extraction layers. Added ethyl acetate-*n*-butanol (4:1, v/v) solvent for extraction for 3 times, and the added volumes were 300, 200, and 150, respectively. After merging extraction layers, the extraction of ginseng berry extraction IV (GBE-4) was obtained. Add ethyl acetate-*n*-butanol (10:9, v/v) solvent, extracted for 3 times, with the volume of 300, 200, and 150 ml, respectively, and combined to obtain the extraction of ginseng berry extraction V (GBE-5). Also, *N*-butanol was added for extraction for 3 times, and each time, the volume was the same as that of the remaining aqueous solution. After the combination, the extraction of ginseng berry extraction VI (GBE-6) was obtained. Finally, the GBE-6 fraction was back extracted with distilled water for 3 times, and the amount of each time was 1/2 of the volume of *n*-butanol. After converged, the ginseng berry stripping VII (GBE-7) was obtained. Weighed 6 parts of GBE, extracted 6 times in parallel with the above method, concentrated each extraction layer under reduced pressure, and froze dry to obtain the corresponding polar fractions; GBE-1 and GBE-2 fractions were liposoluble constituents, which were not used in subsequent experiments.

### Screening of Hypoglycemic Activity of HepG2 Cell Insulin Resistance Model

#### Cell Culture

The HepG2 cells were cultured in RPMI 1640 completed medium (SH30809-01, Hyclone, Logan, UT, USA), containing 10% fetal bovine serum (FBS, Gibco, Grand Island, NY, USA) and 1% penicillin-streptomycin (BBI life Sciences, Shanghai, China) in a 5% CO_2_ humidified atmosphere at 37°C. Took the cells in logarithmic growth period for the experiment.

#### Effects of GBE Fractions on HepG2 Cells Proliferation

The HepG2 cells (5 × 10^4^ cells·ml^−1^) were seeded in 96-well cell culture plates,100 μl per hole, and cultured in 5% CO_2_ incubator at 37°C for 24 h. Then they are divided into 3 groups: Blank group, control group (CON group), and experimental groups. The blank group had only completed medium without cells and drugs, the CON group was normal cultured cells by completed medium without drugs, and experimental groups were normal cultured cells added with GB extraction fractions GBE-3, GBE-4, GBE-5, GBE−6, and GBE-7, respectively, which also dissolved in the completed medium. The concentrations of each fraction were 1,000, 500, 250, 125, 62.5, and 36.25 μg·ml^−1^, respectively. All groups and fractions set six multiple holes with 100 μl per hole. All complete medium contained 0.1% dimethyl sulfoxide (DMSO). Then cultured for 24 h, and the medium was sucked out, add another 100 μl basal-medium as well as 10 μl CCK-8 solution to each hole, incubated for another 2 h, and then determined the absorbance value at 450 nm.

#### Establishment of Insulin Resistance Model of HepG2 Cells

The insulin resistance model of HepG2 cells was established by palmitic acid (PA, Solarbio, Beijing, China). A 100 mmol·L^−1^ palmitic acid (PA) solution was prepared with absolute ethanol; 0.1 mol L^−1^ NaOH solution and 10% BSA solution were prepared with ultrapure water. Mixed the PA solution with NaOH solution (1:1 v/v) by water bath at 70°C for 30 min, the mixed solution was then mixed with 10% BSA solution (1:19 v/v), vortex for 30 s, and water bath at 55°C for 15 min to prepare PA working fluid (2.5 mmol·L^−1^). Diluted the PA working fluid with complete medium at the concentrations of 1, 0.75, 0.5, and 0.25 mmol·L^−1^, respectively, and filtered. HepG2 cells (5 × 10^4^ cells·ml^−1^, dissolved in completed medium) were seeded in 96-well microtiter plates, and set up the following three groups: Blank group (only complete medium), normal group (cells with complete medium), and model group, respectively. The model group was added with concentrations of 1, 0.75, 0.5, and 0.25 mmol·L^−1^ PA working fluid, respectively. Also, six multiple holes were set with 100 μl per hole in every groups. After incubation at 37°C for 24 h, sucked out the culture-medium of each group, washed it with phosphate buffered saline (PBS, Gibco, Carlsbad, CA, USA) for 3 times, and added 100 μl basal-medium in each group, continued to cultivate for 24 h; then detected the glucose content in the medium, and calculated the glucose consumption (GC) of cells in each group.

#### Effects of GBE Fractions on Glucose Consumption in Cells Model

The HepG2 cells (5 × 10^4^ cells·ml^−1^) were seeded on 96-well cell culture plates with 100 μl per hole. Set up a blank group (only blank group had no cells), a normal group, a model group, a positive group, and an experimental group, with six multiple holes in each group. Discarded the original medium, continued to add complete medium to the blank group and normal group, and add 0.25 mmol·L^−1^ PA working fluid to the model group, positive group, and experimental group, 100 μl per hole in each group. Then, cells were incubated for 24 h to establish the insulin resistance model of HepG2 cells. After the experimental model was established, the medium of each group was sucked out and washed with PBS for 3 times. Then the blank group, normal group, and model group were added with complete medium which had no drugs. The positive group was added with 165 μg·ml^−1^ concentration of metformin which dissolved in complete medium. The experimental group was added with complete medium containing the GB extracted fractions GBE-3, GBE-4, GBE-5, GBE−6, and GBE-7, the concentrations of each extracted fraction were 200, 100, 50, 25, 12.5, and 6.25 μg·ml^−1^, respectively. Every medium contained 0.1% DMSO. Each group was incubated for another 24 h, then the medium was discarded, washed with PBS for 3 times, add 100 μl basal-medium in each group, incubated again in 5% CO_2_ incubator at 37°C for 24 h. Then the glucose content in each group were measured. Cell GC = Glucose content of blank group – Glucose content of corresponding group.

#### Cell Viability Assay

After the glucose content of each group was detected, 10 μl CCK-8 reagent was added to each well of 96-well cell culture plates, which was then cultured for 2 h, and the absorbance value at 450 nm was measured to calculate the cell survival rate. The cell survival rate = [(*A* – *C*) /(*B* – *C*)] × 100%, where *A* was the absorbance value of the experimental group, *B* was the absorbance value of the normal group, and *C* was the absorbance value of the blank group.

### Animal Experiments

#### Animal Feeding and Grouping

A 7-week-old adult male Wistar rats (weighing 190 ± 10 g) were supplied by the Experimental Animal Center of Jilin University (Changchun, Jilin, China). All rat experimental procedures were performed in accordance with the Regulations for the Administration of Affairs Concerning Experimental Animals approved by the State Council of People's Republic of China. Rats were raised in a standard SPF barrier environment. The conditions were constant temperature of 23 ± 2°C, relative humidity of 40–80%, alternating day and night light cycle of 6:00 a.m. to 6:00 p.m., and the rats were free to eat and drink. After 7 days of environmental adaptation, 8 rats were randomly selected as the CON group, which was fed with ordinary diet during the whole experiment. The other rats were fed with HFD, which were consisting of 10.0% lard, 20.0% sucrose, 2.5% cholesterol, 1.0% sodium cholate, and 66.5% pulverized standard rat pellet (7). After 8 weeks of HFD feeding, the rats were intraperitoneally injected with freshly prepared STZ (40 mg·kg^−1^ b.w.) dissolved in citric acid buffer (0.1 mol·L^−1^, pH 4.3–4.5). The rats in CON group were only injected with citric acid buffer. After 7 days, fasting blood glucose was measured after fasting for 12 h. The blood glucose value ≥7.8 mmol L^−1^, accompanied by symptom of polyuria and polydipsia, was regarded as successfully established T2DM rats. Also, 24 T2DM rats were randomly divided into three groups (*n* = 8), including GBE-5 group, which was fed HFD diet, and intragastrically administered with 200 mg·kg^−1^ (b.w.) ginseng berry GBE-5 fraction dissolved in saline once daily. The metformin (MET) group was fed with HFD diet, and intragastrically administered metformin dissolved in saline once daily, with a therapeutic dose of 100 mg · kg^−1^ (b. w.). The T2DM group was fed with HFD diet and intragastrically administered with saline once daily. The CON group was fed with normal diet and intragastrically administered with saline once daily. The blood glucose value of rats after fasting for 12 h was measured every 7 days; the body weight (b.w.), food consumption, and water consumption within 24 h were measured every 4 days.

#### Serum Sample Collection and Preparation

After 30 days of continuous intervention, the rats fasted for 12 h, and were anesthetized by intraperitoneal injection of 10% chloral hydrate (3 ml/kg b. w.) for 15 min, and blood was collected from the abdominal aorta of the rats. After the plasma was left at room temperature for 60 min, centrifuged at 3,000*g* and 4°C for 15 min, it was immediately put into liquid nitrogen and finally stored in the refrigerator at −80°C. The serum samples were thawed at 4°C before analysis, and 250 μl of serum mixed with 750 μl chromatographic methanol (1:3 v/v, 4°C), vortex for 30 s, and then centrifuged at 12,000 rpm for 15 min at 4°C. Take the supernatant, blow dry with nitrogen, add 1.0 l of ultrapure water for re-dissolution (4°C), vortex for 15 s, centrifuged again at 12,000 rpm for 15 min at 4°C, and the supernatant was filtered through 0.22 μm membrane filter before UHPLC–MS detection.

### UHPLC-MS Analysis

An analysis was performed using UHPLC-ESI-Q-Exactive Orbitrap/MS system. The UHPLC was equipped with UltiMate 3000 system (Thermo Fisher Scientific, Dionex, Sunnyvale, CA, USA) coupled with Golden C_18_ column (2.1 × 50 mm, 1.9 μm, Thermo Fisher Scientific, San Jose, CA, USA) maintained at 35°C. The automatic sampler and sample chamber temperature was 4°C, sample injection volume was 2.0 μl, the flow rate was 0.3 ml/min, the mobile phase A was 0.1% formic acid in water, and phase B was 100% acetonitrile. The chromatographic conditions of rat serum were: 0–1 min, 3% phase B, 1–16 min, 3–70% phase B, 16–18 min, 70% phase B, 18–25 min, 70–90% phase B, 25–26 min, 90–100% phase B, 26–30 min, 100% phase B. The chromatographic conditions of GBE fractions were: 0–20 min, 25–65% phase B, 20–25 min, 65–100% phase B, 25–30 min, 100% phase B.

The MS detection was performed on Q-Exactive orbitrap MS (Thermo Fisher Scientific, USA), for which the source parameters were set as follows: Sweep gas flow was 0.175 Mpa, sheath gas flow was 6.125 Mpa, and auxiliary gas flow was 2.625 Mpa. The capillary temperature was 320°C, the capillary voltage was 3.5 kV, and the auxiliary gas heater temperature was 350°C. The electrospray ionization source in both positive (ESI+, pos) and negative (ESI–, neg) ion modes was used, and the scanning range were m/z 66.7 ~ 1,000 (rat serum) and m/z 133.4 ~ 2,000 (GBE fractions). The scan mode was Full MS with dd-MS^2^, Top *N* = 8, of which the resolution was 17,500 (date dependent-MS^2^) and 70,000 (Full MS), respectively, and the collision energy was 10, 20, and 40%, when it was dd-MS^2^ mode. The instrument was able to meet the quality accuracy of pos/neg and MS/MS^2^ cycling. Our selected acquisition mode improved the efficiency of acquisition, and also met the requirements of the established secondary MS^2^ identification efficiency. The data were recorded and analyzed using the Xcalibur software (Version 2.2.42, Thermo Fisher Scientific, USA).

### Quality Control Sample Preparation

To test the stability and reproducibility of the system, quality control (QC) samples of rat serum and GBE fractions were prepared, respectively. Took a small part of the same volume from all samples, mixed it and treated it according to the treatment method of corresponding samples to obtain QC samples of rat serum and GBE fractions, respectively. Before the experiment, QC samples were tested 6 times to balance the UHPLC–MS analysis system, and then QC samples and blank samples (75% acetonitrile in water) were tested once between every 6 samples in the sample detection sequence.

### Multivariate Data Processing and Analysis

The raw data of rat serum was obtained from Dionex^TM^, Chromeleon^TM^ 6.8, and Thermo Xcalibur, which was then imported into Progenesis QI v 2.3 software (Newcastle, UK) and used for deconvolution, calibrate the retention time (RT), peak alignment, and normalization. Combined the positive and negative ion data into a data matrix and saved as Excel file, which was then imported to the R programming language software (Ropls package v3.6.2) for the principal components analysis (PCA), and observed the overall distribution of each sample and the stability of the UHPLC–MS analysis process. Univariate statistical analysis was carried out by Stats software package, and ggplot2 package was used to get the volcano plot, which could visualize the *p*-value and the fold change (FC) value. Metabolite meeting *p* < 0.05 as well as FC ≥ 1.5 or FC ≤ 0.67 was usually considered to be differential metabolites.

### Differential Metabolites Identification and Pathway Analysis

Metabolomics databases such as human metabolome database (HMDB) (http://www.hmdb.ca/), Lipidmaps v2.3 (http://www.lipidmaps.org), and METLIN (https://metlin.scripps.edu/) were screened and identified the matched metabolites, when the product ion mass spectra and MS/MS fragment ion suited with the structural information in the databases. The KEGG (https://www.kegg.jp/) database was used for metabolic pathway enrichment analysis of potential differential metabolites.

## Results and Discussion

### Analysis of GBE Fractions by UHPLC–MS

#### The PCA Analysis

**Figure 1:**
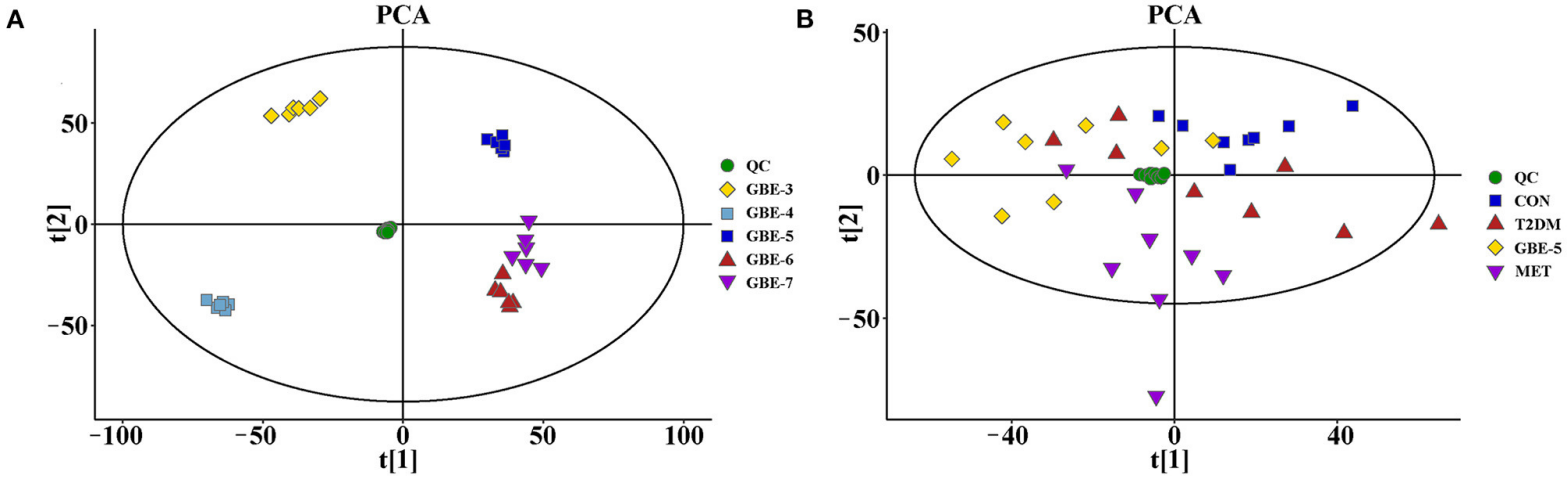
The principal component analysis (PCA) score plots of GBE fractions **(A)** and rat serum samples **(B)**.

In order to verify the stability and reliability of the UHPLC-MS and acquired data, PCA analysis was carried out on GBE fractions and its QC samples. As shown in Figure 1A, it could be observed that QC samples showed a good aggregation state in the PCA score plot and approached the coordinate origin, indicating that the experimental condition was in a stable state during the data collection.

#### Analysis of the GB Extract Fractions by UHPLC–MS

**Figure 2:**
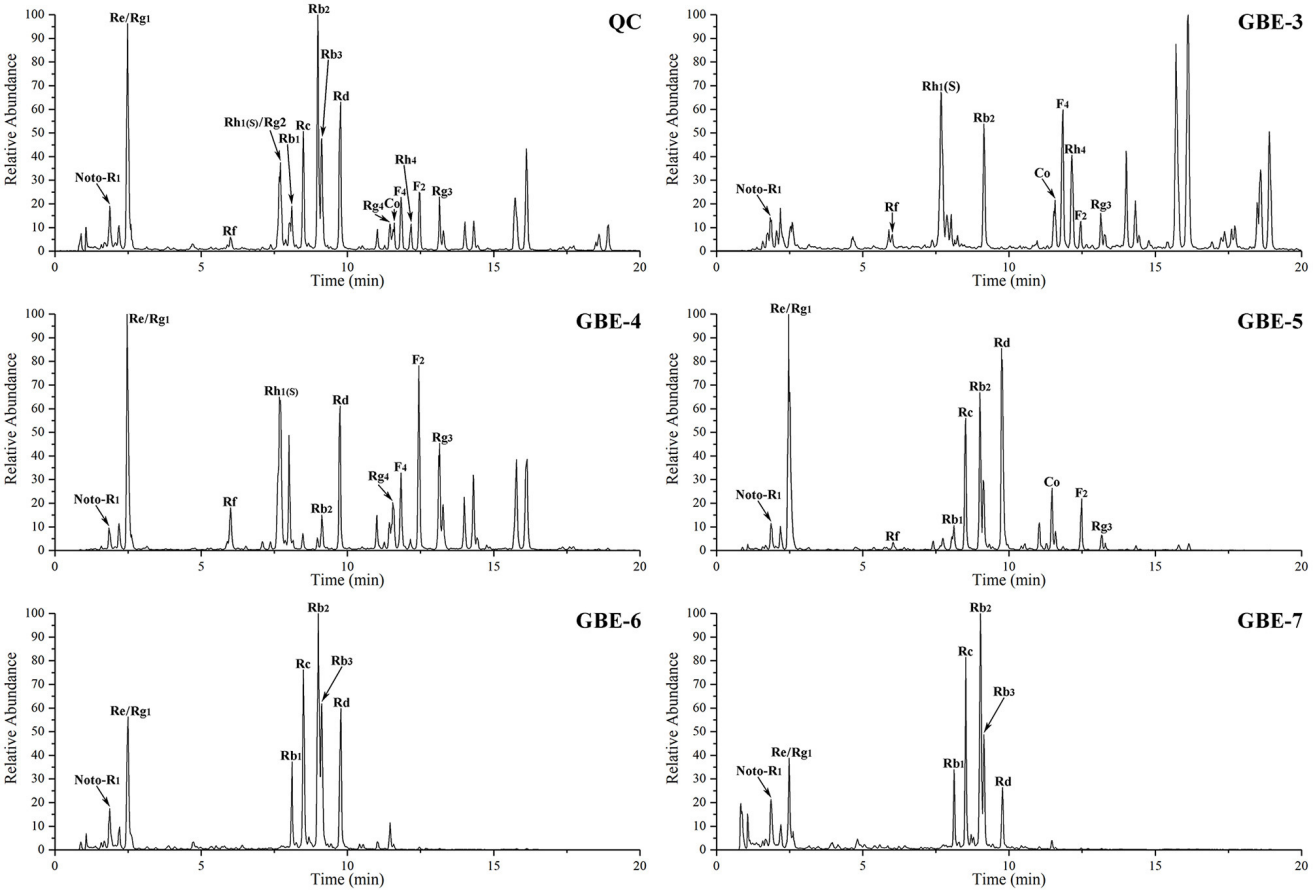
The UHPLC–MS TIC of GBE fraction.

The total ion chromatograms (TIC) of the GBE fractions obtained by UHPLC–MS were shown in Figure 2, in which both were in negative ion mode (ESI-), and the ion peaks in the spectrum were well separated, indicating that the corresponding chromatographic and MS conditions were suitable for samples determination. As the polarity of the seven reagents (or mixed reagents) used in the extraction segmentation process increased in turn, the polarity was petroleum ether < ethyl ether < ethyl acetate–ethyl ether (3:2, v/v) < ethyl acetate-*n*-butanol (4:1, v/v) < ethyl acetate-*n*-butanol (10:9, v/v) < *n*-butanol < distilled water, respectively. Therefore, the polarity of the corresponding extraction fractions GBE-1 to GBE-7 also increased in turn, which could be seen from the TIC that the RT (min) of the active components in GBE-3 to GBE-7 fraction gradually moved to the left (GBE-1 and 2 fractions were discarded), indicating that the polarity of the main active components of each fraction gradually increased, suggesting that the solvent extraction method could segmented the GBE well. Weighed 30 g of GBE, respectively; prepared the extract fractions in parallel for 6 times; and dried them to constant weight. The final average weight of each fraction was 0.51 ± 0.03 g for GBE-3, 6.05 ± 0.08 g for GBE-4, 12.31 ± 0.18 g for GBE-5, 5.76 ± 0.16 g for GBE-6, and 1.37 ± 0.09 g for GBE-7 fraction, respectively. It could be seen that the main active components of GBE were concentrated in GBE-4 to GBE-6 fractions. The main component of GBE-4 was 20(*S*)-protopanaxatriol, while the main component of GBE-5 and GBE-6 was 20(*S*)-protopanaxadiol. The result was expressed as mean ± SD.

### Screening of Hypoglycemic Active Fraction From GBE

#### Effects of GBE Fractions on the Proliferation of HepG2 Cells

The effect of GBE concentration on HepG2 cell proliferation was investigated, it was found that when the drug concentration was ≥ 250 μmol·L^−1^, the cell survival rate of the experimental group was generally less than 50%. Therefore, the administration concentration of cells should be less than 250 μmol·L^−1^. Since six different administration concentrations were set for each fraction of GBE, the final concentrations were set as 200, 100, 50, 25, 12.5, and 6.25 μmol·L^−1^.

#### The Result of HepG2 Cells Insulin Resistance Model Establishment

The HepG2 insulin resistance model was established by PA. It was found that when the concentrations of PA were 1, 0.75, 0.5, and 0.25 mmol·L^−1^, the GC of each model group was significantly lower than that of the normal group (*p* < 0.01), indicating that the HepG2 insulin resistance model was successfully established by PA at each concentration, which was shown in Table 1. Considering the effect of PA concentration on HepG2 cell survival, the smallest PA concentration (0.25 mmol·L^−1^) was selected for modeling, which was also consistent with that reported in the literature (23, 24).

**Table 1:** The effects of four concentrations of PA on glucose consumption of HepG2 cells.

**Groups**	**Contents**	**GC** **(mmol·L^**−1**^)**
Blank group	Only complete medium	14.635 ± 0.775
Normal group	HepG2 cell	5.274 ± 0.369
Model group	HepG2 cell added 1.0 mmol·L^−1^ PA	0.858 ± 0.615^*^
	HepG2 cell added 0.75 mmol·L^−1^ PA	0.992 ± 0.226^*^
	HepG2 cell added 0.5 mmol·L^−1^ PA	1.244 ± 0.549^*^
	HepG2 cell added 0.25 mmol·L^−1^ PA	1.526 ± 0.863^*^

*Data was expressed as mean ± SD*.

* *p < 0.01 vs. normal group*.

#### Effects of GBE Fractions on GC in Insulin Resistance Model

**Figure 3:**
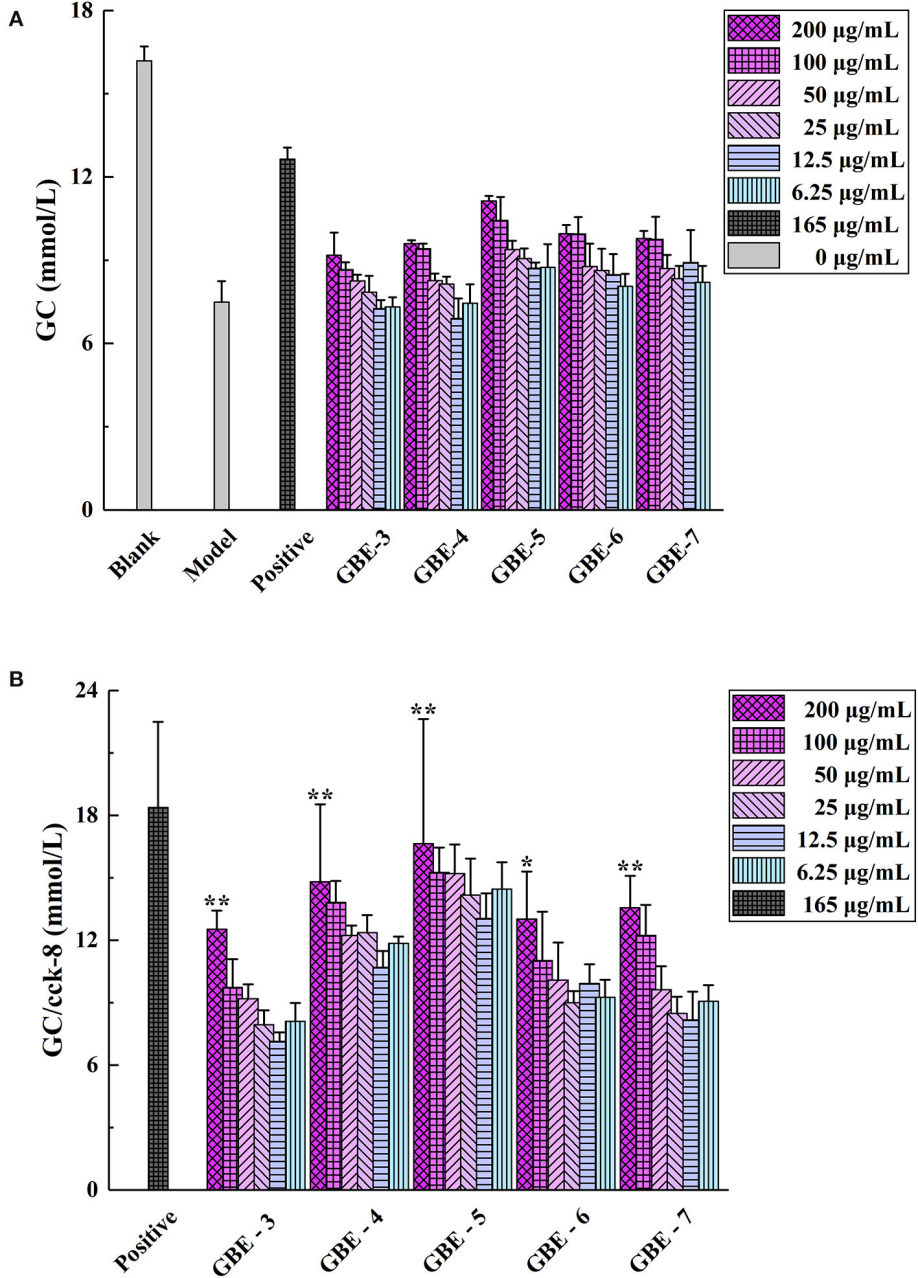
**(A)** Cell GC and **(B)** GC/Cell survival rate (CCK-8). *p < 0.05 vs. model group, ** *p* < 0.01 vs. model group.

The screening results of hypoglycemic activity of GBE fractions were shown in Table 2. It was found that the GC of the cells in the model group was significantly lower than that in the normal group (*p* < 0.01), indicating that the model of insulin resistance of HepG2 cells was successfully established. The GC of GBE fractions in the experimental group were significantly higher than (*p* < 0.05) or very significantly higher than (*p* < 0.01) the model group, suggesting that the drug intervention could improve the GC of HepG2 cell insulin resistance model. The cell GC of the blank group, model group, positive group, and experimental group was shown in Figure 3A. The ratio of GC to cell survival rate (CCK-8) was calculated to eliminate the influence of the number of cells in the group on the GC of each group. The calculation results were shown in Figure 3B. It was found that GBE-5 fraction has better hypoglycemic activity than other extraction fractions, but the hypoglycemic effect was worse than that of the positive group (*p* > 0.05). The main components of GBE-5 fraction were putatively identified by the accurate mass measurement and MS^2^ fragment ions obtained by UHPLC–MS, as well as the database searching. The molecular MS error of all identified metabolites was less than 10 ppm. The compounds of GBE-5 were final identified by comparing the RT, accurate mass, and MS/MS spectra of authentic standards. The analyzed data were shown in Table 3.

**Table 2:** The effect of GBE fractions on GC of insulin resistant HepG2 cells.

**Groups**	**Drug Dosage (μg·ml^**−1**^)**	**GC** **(mmol·L^**−1**^)**	**CCK-8 (A 450)**	**GC/CCK-8** **(mmol·L^**−1**^)**
Blank group	0	16.187 ± 0.517	-	-
Normal group	0	14.323 ± 0.379	1.021 ± 0.077	14.111 ± 1.090^*^
Model group	0	7.487 ± 0.755	0.914 ± 0.041	7.707 ± 0.983^▴^
Positive group	165	12.646 ± 0.417	0.731 ± 0.195	18.375 ± 4.113
GBE-3 fraction	200	9.185 ± 0.808	0.735 ± 0.058	12.526 ± 0.890^**^
	100	8.662 ± 0.269	0.902 ± 0.087	9.727 ± 1.361
	50	8.258 ± 0.224	0.904 ± 0.083	9.192 ± 0.692
	25	7.849 ± 0.590	0.994 ± 0.086	7.934 ± 0.699
	12.5	7.255 ± 0.303	1.020 ± 0.071	7.136 ± 0.438
	6.25	7.319 ± 0.342	0.913 ± 0.097	8.103 ± 0.876
GBE-4 fraction	200	9.596 ± 0.128	0.701 ± 0.212	14.800 ± 3.725^**^
	100	9.410 ± 0.189	0.685 ± 0.057	13.817 ± 1.030
	50	8.264 ± 0.254	0.677 ± 0.033	12.223 ± 0.463
	25	8.143 ± 0.260	0.663 ± 0.061	12.359 ± 0.848
	12.5	6.897 ± 0.730	0.649 ± 0.090	10.689 ± 0.787
	6.25	7.454 ± 0.679	0.629 ± 0.056	11.853 ± 0.322
GBE-5 fraction	200	11.135 ± 0.184	0.769 ± 0.292	16.637 ± 5.986^**^
	100	10.437 ± 0.846	0.688 ± 0.072	15.248 ± 1.202
	50	9.381 ± 0.324	0.622 ± 0.055	15.204 ± 1.398
	25	9.060 ± 0.368	0.649 ± 0.081	14.163 ± 1.755
	12.5	8.708 ± 0.213	0.674 ± 0.072	13.039 ± 1.207
	6.25	8.747 ± 0.830	0.607 ± 0.053	14.458 ± 1.290
GBE-6 fraction	200	9.955 ± 0.314	0.790 ± 0.145	13.011 ± 2.278^*^
	100	9.943 ± 0.608	0.964 ± 0.288	11.013 ± 2.349
	50	8.769 ± 0.827	0.903 ± 0.209	10.078 ± 1.811
	25	8.635 ± 0.784	0.965 ± 0.115	8.995 ± 0.557
	12.5	8.482 ± 0.744	0.859 ± 0.078	9.918 ± 0.921
	6.25	8.059 ± 0.446	0.882 ± 0.124	9.250 ± 0.850
GBE-7 fraction	200	9.778 ± 0.282	0.730 ± 0.081	13.553 ± 1.545^**^
	100	9.754 ± 0.808	0.802 ± 0.056	12.233 ± 1.458
	50	8.700 ± 0.492	0.916 ± 0.109	9.619 ± 1.135
	25	8.337 ± 0.460	0.991 ± 0.100	8.480 ± 0.799
	12.5	8.920 ± 1.168	1.103 ± 0.123	8.174 ± 1.350
	6.25	8.206 ± 0.583	0.907 ± 0.025	9.061 ± 0.784

*Data was expressed as mean ± SD*.

* *p < 0.05 vs. model group*.

** *p < 0.01 vs. model group*.

▴ *p < 0.01 vs. GBE-5 fraction group*.

**Table 3:** Identification of compounds in GBE-5 fraction from ginseng berry total ginsenosides.

**No**.	**Compounds**	**Formula**	**Accurate mass (m/z)**	**Measured mass (m/z)**	**Adducts**	**MS^**2**^ fragment ions (Mass accuracy <10 ppm)**
1	Notoginsenoside R_1_	C_47_H_80_O_18_	932.5345	931.5268	[M-H]^−^	781.4741, 751.4636, 637.4318, 619.4213, 149.0450
2	Ginsenoside Rg_1_	C_42_H_72_O_14_	800.4922	845.4904	[M+FA-H]^−^	781.4753, 637.4319,619.4216,457.3685, 179.0559
3	Ginsenoside Re	C_48_H_82_O_18_	946.5501	991.5483	[M+FA-H]^−^	781.4740, 637.4319, 619.4215, 279.1085, 163.0612
4	Ginsenoside Rf	C_42_H_72_O_14_	800.4922	799.4846	[M-H]^−^	619.4213, 475.3792, 457.3685, 295.1032, 179.0569
5	Ginsenoside Rb_1_	C_54_H_92_O_23_	1108.6029	1107.5953	[M-H]^−^	945.5426, 783.4899, 691.4426, 341.1086, 179.0553
6	Ginsenoside Rc	C_53_H_90_O_22_	1078.5924	1077.5856	[M-H]^−^	969.5436, 897.5224, 735.4696, 179.0571, 56.9989
7	Ginsenoside Rb_2_	C_53_H_90_O_22_	1078.5924	1077.5853	[M-H]^−^	897.5228, 753.4799, 645.4387, 149.0466, 131.0351
8	Ginsenoside Rb_3_	C_53_H_90_O_22_	1078.5924	1077.5846	[M-H]^−^	945.5089, 621.4128, 293.0719, 149.0389, 83.4633
9	Ginsenoside Rd	C_48_H_82_O_18_	946.5501	991.5485	[M+FA-H]^−^	783.4883, 621.4351, 529.3881, 295.1021
10	Ginsenoside F_2_	C_42_H_72_O_13_	784.4973	783.4898	[M-H]^−^	621.4358, 603.4253, 529.3887, 499.3779, 149.0463
11	Ginsenoside Rg_3_	C_42_H_72_O_13_	784.4973	783.4893	[M-H]^−^	603.4257, 459.3846, 441.3746, 179.0567

### Anti-diabetic Effects of GBE-5 Fraction in T2DM Rats

**Figure 4:**
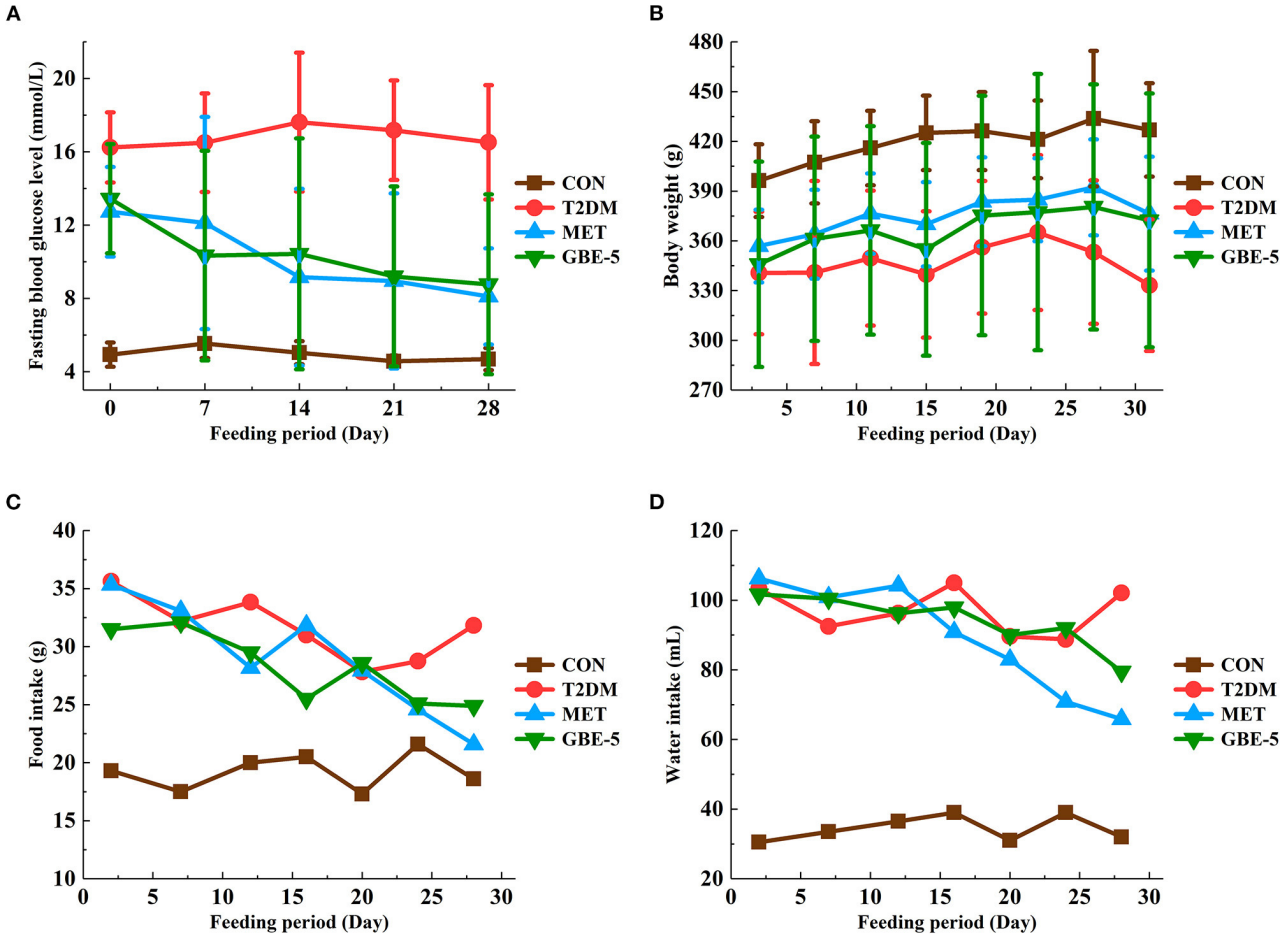
The effect of GBE-5 fraction on the fasting blood glucose level **(A)**, body weight **(B)**, food **(C)**, and water **(D)** intake in T2DM rats.

A rat model of T2DM induced by HFD and STZ was used to study the hypoglycemic effect of GBE-5 fraction. At the end of the 30-days experiment, it was found that GBE-5 fraction could significantly reduce the fasting blood glucose level in T2DM rats, and decreased from 13.44 ± 2.99 mmol·L^−1^ to 8.77 ± 4.91 mmol·L^−1^ (*p* < 0.05), as shown in Figure 4, food and water intake decreased in MET group and GBE-5 group, and there was no significant difference in body weight among the groups (*p* > 0.05). In the previous studies, it was found that total ginsenosides of GB could reduce the fasting blood glucose of rats by 27.35% (7). On the premise of the same drug dosage and treatment time, the fasting blood glucose of GBE-5 fraction which derived from GB total ginsenosides decreased significantly by 34.75% after 30 days of intervention in T2DM rats, indicating that the hypoglycemic effect of GBE-5 fraction was better than that of total ginsenosides of GB.

### Serum Metabolic Profiling by UHPLC–MS

**Figure 5:**
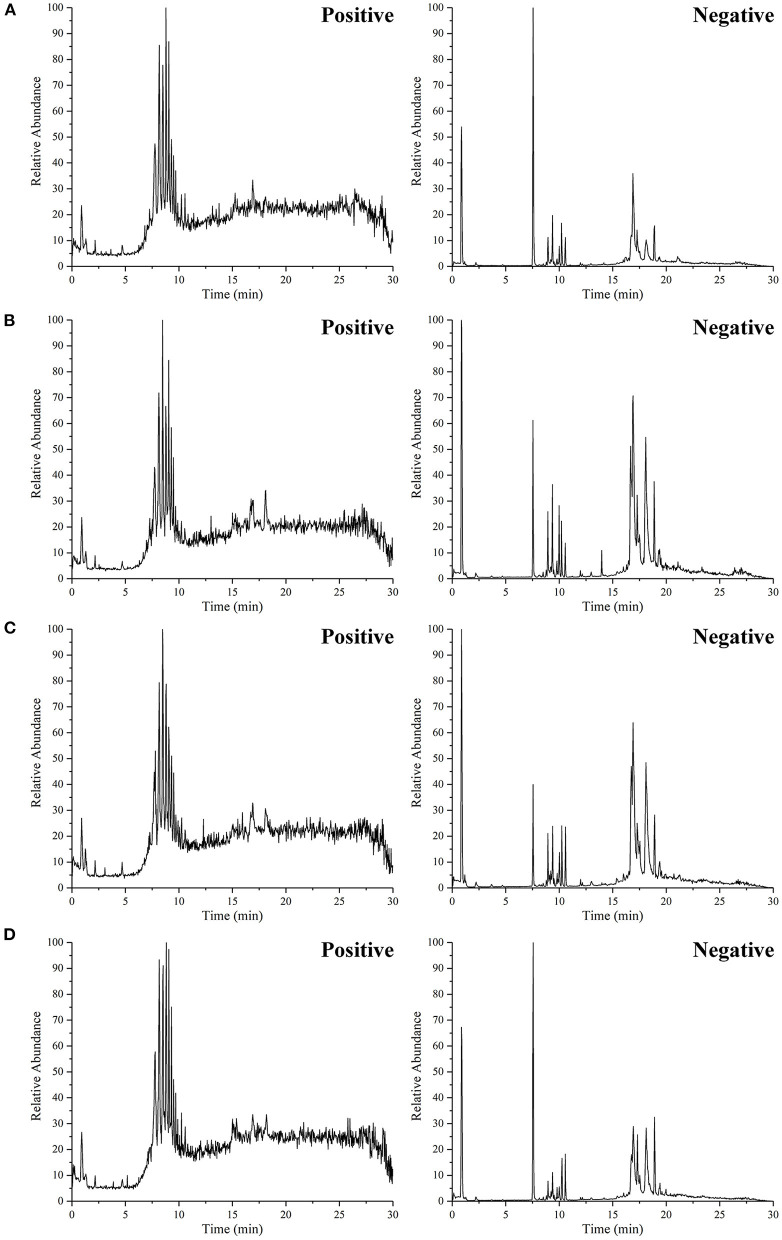
The representative UHPLC–MS TIC of serum from the CON group **(A)**, T2DM group **(B)**, MET group **(C)**, and GBE-5 fraction group **(D)**; the TIC on the left side was positive modes and the right side was negative modes, respectively.

The positive and negative TIC of rat serum samples collected by UHPLC–MS was shown in Figure 5, in which A was CON group, B was T2DM group, C was MET group, and D was GBE-5 group. The ion peaks in the spectrum were well separated, indicating that the UHPLC–MS conditions in this experiment were suitable for sample determination.

### Univariate Statistical Analysis

**Figure 6:**
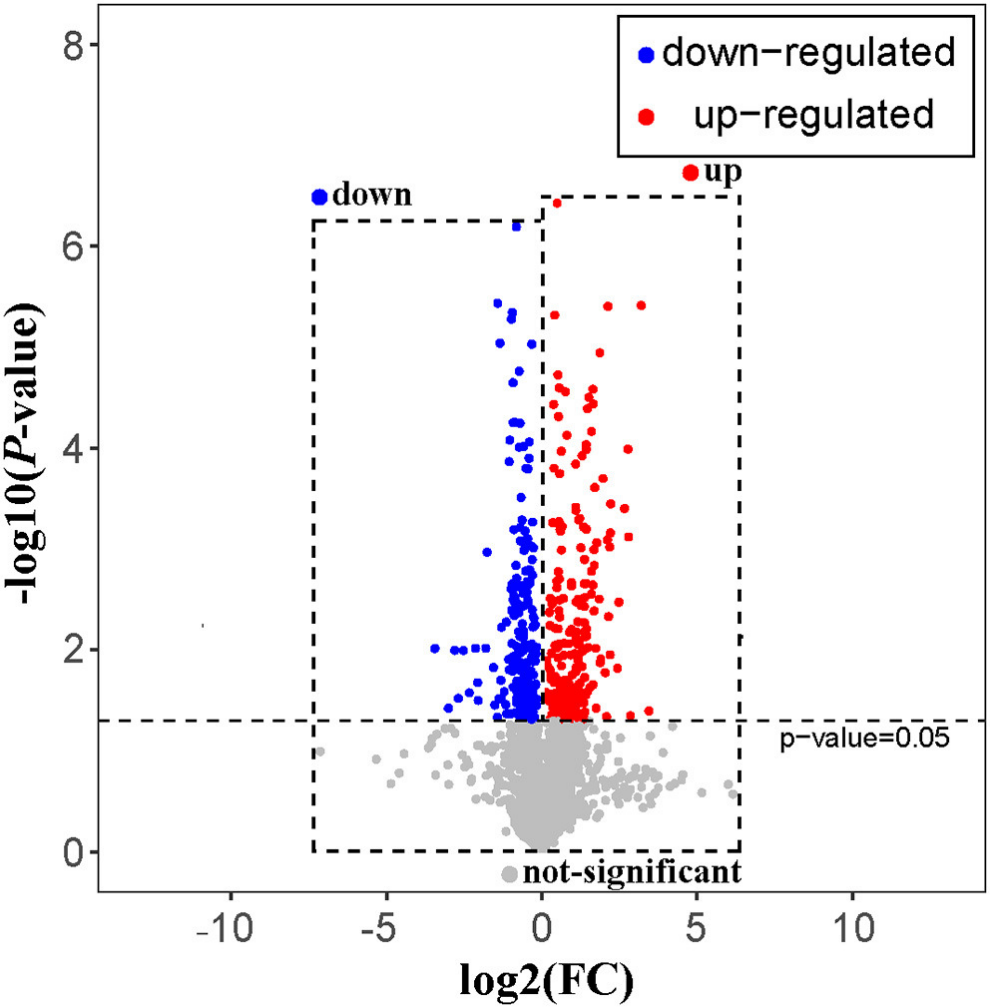
The volcano plot of T2DM vs. GBE-5 fraction group.

The rat serum and its QC samples were analyzed by PCA, as shown in Figure 1B, the QC samples showed a good aggregation state and closed to the coordinate origin, indicating that the acquisition instrument was in a stable state during data collection. Univariate statistical analysis based on UHPLC–MS data was used to screen the differential metabolites in rat serum after the 30-days intervention of GBE-5 fraction. Univariate analysis often used the FC value to represent the regulation multiple of metabolites between the two comparison groups. It was generally considered that compounds with FC value ≥ 1.5 or ≤ 0.67 and *p* < 0.05 were the differential metabolites between groups, which could be visualized by volcano plot, as shown in Figure 6, the red dots represented the significantly up-regulated differential metabolites (log_2_FC > 0, *p* < 0.05), the blue dots represented the significantly down-regulated differential metabolites (log_2_FC <0, *p* < 0.05), and the gray dots represented the metabolites with no significant regulation (*p* > 0.05).

### Identification of Metabolites and KEGG Pathway Enrichment Analysis

**Figure 7:**
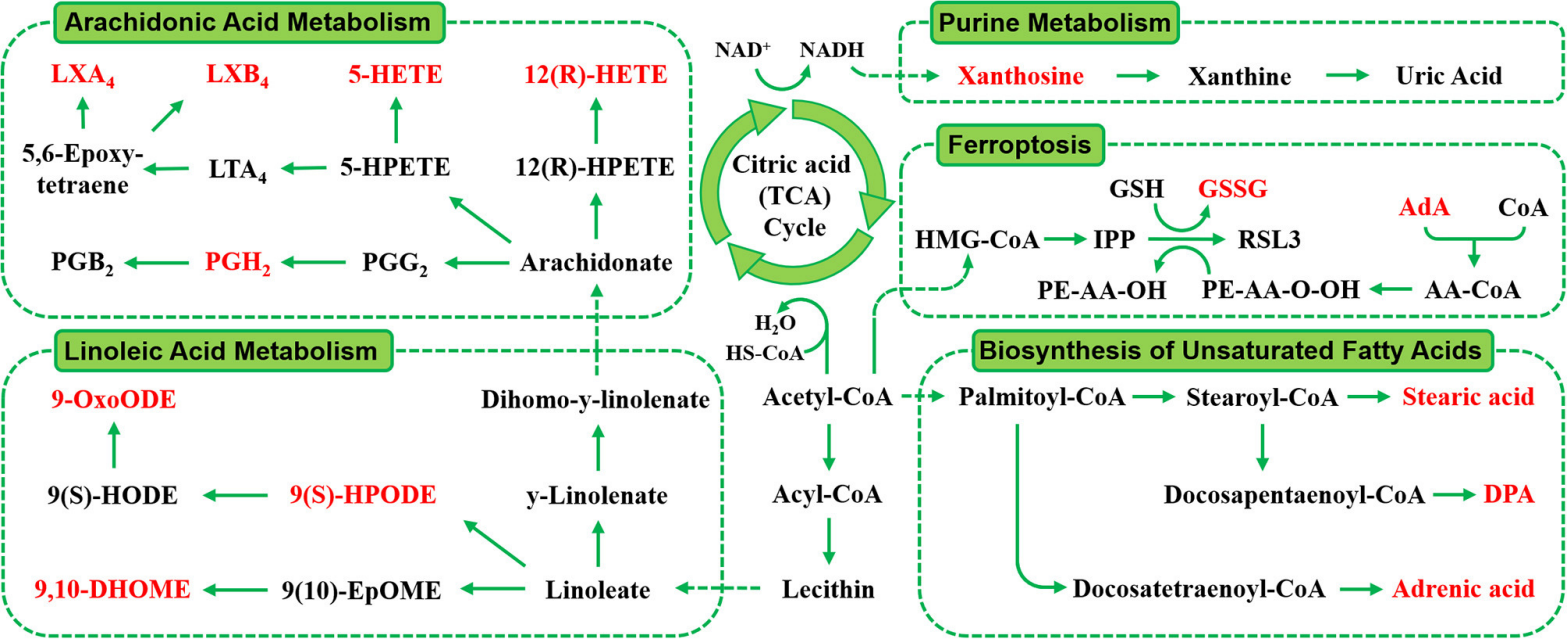
The correlation networks between main differential metabolites with corresponding metabolic pathways.

The RT, accurate mass spectra and MS/MS spectra of the differential compounds screened by univariate statistical analysis were imported into the metabolomics database, the data information of the standard in the database was compared, the mass error of all metabolites was less than 10 ppm, so the compounds were qualitatively identified. There were 66 compounds screened as differential metabolites in the GBE-5 *vs*. T2DM group. Then the KEGG online database was used to pathway enrichment analysis, a total of 18 differential metabolites could be enriched in the KEGG metabolic pathway. The differential metabolites information and metabolic pathway enrichment results were shown in Tables 4, 5, respectively. It was found that the four main metabolic pathways involved in regulation maybe the potential regulatory mechanism for the anti-diabetic effect of the GBE-5 fraction. Among them, five differential metabolites: 12(*R*)-HETE, 5-HETE, Lipoxin A4, Lipoxin B4, and PGH2 were involved in AA metabolism, three differential metabolites: 9(*S*)-HPODE, 9,10-DHOME, and 9-OxoODE were involved in linoleic acid (LA) metabolism, three differential metabolites: Adrenic acid (AdA), docosapentaenoic acid (DPA), and stearic acid were involved in the biosynthesis of unsaturated fatty acids, and two differential metabolites: Oxidized glutathione (GSSG) and AdA were involved in ferroptosis. These metabolic pathways were all widely linked to T2DM, and the correlation networks of differential metabolites mentioned discussed was shown in Figure 7.

**Table 4:** The identification results of differential metabolites in T2DM vs. GBE-5 fraction group in serum.

**No**.	**Mode**	**Metabolite**	**Formula**	**Adduct**	***t*** **_R_/min**	**Measured m/z**	**MS^**2**^ fragment ions**	**Error (ppm)**	**FC**	***p***
1	Pos	Metanephrine	C_10_H_15_NO_3_	[M+K]^+^	0.85	236.0676	180.1017, 149.0595, 125.0595	−3.9	1.71	0.00003
2	Pos	L-Proline	C_5_H_9_NO_2_	[M+Na]^+^	1.02	138.0527	98.0608, 87.0456, 70.0659	1.3	2.17	0.00335
3	Neg	12(*R*)-HETE	C_20_H_32_O_3_	[M-H]^−^	17.29	319.2266	275.2368, 179.1066, 111.1168	−2.6	2.88	0.00003
4	Neg	5-HETE	C_20_H_32_O_3_	[M-H_2_O-H]^−^	18.84	301.2164	301.2160, 257.2261, 87.0438	−2.7	1.75	0.03753
5	Neg	9(*S*)-HPODE	C_18_H_32_O_4_	[M-H]^−^	15.96	311.2220	277.2165, 233.2267, 169.1226	−2.4	3.23	0.00102
6	Neg	9,10-DHOME	C_18_H_34_O_4_	[M-H]^−^	15.21	313.2378	295.2273, 251.2375, 171.1021	−2.1	2.15	0.00041
7	Neg	9-OxoODE	C_18_H_30_O_3_	[M-H]^−^	16.14	293.2118	275.2012, 249.2220, 185.1179	−1.4	2.41	0.00097
8	Neg	ADA	C_22_H_36_O_2_	[M-H]^−^	20.22	331.2632	313.2527, 287.2734, 271.2421	−3.2	1.70	0.01985
9	Neg	Dihydroneopterin triphosphate	C_9_H_16_N_5_O_13_P_3_	[M-H]^−^	16.91	493.9876	408.9601, 238.8909, 158.9246	−1.4	1.68	0.03974
10	Neg	DPA	C_22_H_34_O_2_	[M-H]^−^	19.96	329.2475	311.2369, 269.2264	−3.3	2.27	0.00316
11	Neg	Lipoxin A_4_	C_20_H_32_O_5_	[M-H]^−^	14.71	351.2163	333.2057, 217.1583, 115.0387	−3.1	2.82	0.00948
12	Neg	Lipoxin B_4_	C_20_H_32_O_5_	[M-H]^−^	14.94	351.2162	315.1951, 203.1063, 129.0906	−4.1	2.07	0.02378
13	Neg	LysoSM(d18:1)	C_23_H_50_N_2_O_5_P^+^	[M-H_2_O-H]^−^	19.10	446.3280	124.9996, 104.1068	0.1	2.07	0.03470
14	Neg	GSSG	C_20_H_32_N_6_O_12_S_2_	[M-H_2_O-H]^−^	14.09	593.1366	352.0662, 306.0785, 242.0801	4.0	1.67	0.02512
15	Neg	PGH_2_	C_20_H_32_O_5_	[M-H]^−^	13.05	351.2163	333.2057, 225.1118, 99.0801	−4.0	2.47	0.01466
16	Neg	Stearic acid	C_18_H_36_O_2_	[M-H]^−^	20.74	283.2637	265.2526, 239.2734, 155.1795	−1.9	2.05	0.03708
17	Neg	Testosterone	C_19_H_28_O_2_	[M-H]^−^	13.22	287.2013	231.1746, 189.1276, 135.0807	−1.3	2.08	0.05293
18	Neg	Xanthosine	C_10_H_12_N_4_O_6_	[M-H]^−^	1.97	283.0679	151.0251, 133.0145, 108.0193	−1.7	1.85	0.03535

**Table 5:** Enriched KEGG pathway of differential metabolites in T2DM vs. GBE-5 fraction group.

**No**.	**Metabolite**	**Compound ID**	**KEGG**	**Annotation**
1	Metanephrine	HMDB0004063	C05588	Tyrosine metabolism
2	L-Proline	HMDB0000162	C00148	Protein digestion and absorption/Arginine and proline metabolism
3	12(*R*)-HETE	HMDB0062287	C14822	AA metabolism
4	5-HETE	HMDB0011134	C04805	AA metabolism
5	9(*S*)-HPODE	HMDB0006940	C14827	LA metabolism
6	9,10-DHOME	HMDB0004704	C14828	LA metabolism
7	9-OxoODE	HMDB0004669	C14766	LA metabolism
8	ADA	HMDB0002226	C16527	Ferroptosis/Biosynthesis of unsaturated fatty acids
9	Dihydroneopterin triphosphate	HMDB0000980	C04895	Folate biosynthesis
10	DPA	HMDB0006528	C16513	Biosynthesis of unsaturated fatty acids
11	Lipoxin A_4_	HMDB0004385	C06314	AA metabolism /Toxoplasmosis
12	Lipoxin B_4_	HMDB0005082	C06315	AA metabolism
13	LysoSM (d18:1)	HMDB0006482	C03640	Sphingolipid metabolism
14	GSSG	HMDB0003337	C00127	Ferroptosis /Thyroid hormone synthesis/Glutathione metabolism
15	PGH_2_	HMDB0001381	C00427	AA metabolism /Oxytocin signaling pathway/Platelet activation /Retrograde endocannabinoid signaling
16	Stearic acid	HMDB0000827	C01530	Biosynthesis of unsaturated fatty acids /Fatty acid biosynthesis
17	Testosterone	HMDB0000234	C00535	Endocrine resistance /Prostate cancer
18	Xanthosine	HMDB0000299	C01762	Caffeine metabolism

The metabolism of AA, LA, and biosynthesis of unsaturated fatty acids belongs to lipid metabolism. The LA was a kind of unsaturated fatty acids, due to the substantial increase in vegetable oils consumption, the intake of LA also increased greatly, and dietary fatty acids were responsible for the development and occurrence of many chronic diseases, such as obesity and diabetes (25). The LA was the direct precursor of AA) the conversion efficiency of LA into AA in human body was low, they were linked through metabolism. It was reported that AA and LA could be used as predictive markers for gestational diabetes mellitus (26), AA and its derivatives played a key role in the occurrence and development of obesity and T2DM, and played a diverse and partly contrasting roles in the pathogenesis of T2DM. Therefore, the study of AA as well as LA metabolism may help to identify novel targets for the treatment of T2DM and its associated complications (27).

Wang et al. (28) used proteomics to study the differential abundant proteins associated with diabetes in hamster liver. Combined with enrichment analysis of KEGG pathway, found that AA metabolism, LA metabolism, and bile acid (BA) metabolism in diabetic hamsters were affected. Our previous study had also found that GB exerted anti-diabetic effect by regulating AA and BA metabolism (7). However, in this study, the GBE-5 fraction was not involved in the regulation of BA metabolism pathway, which suggested that the screened GBE-5 fraction may not contained all of the GB active substances with anti-diabetic activity. Although the *in vivo* hypoglycemic activity experimental result of GBE-5 showed that its hypoglycemic effect was better than GB and other GBE fractions, the segmentation of GB by polar extraction method had its limitations, which cannot effectively collect all the pharmacodynamic material bases in GB. In the next experiment, the combination could be carried out according to the actual content of several main ginsenosides in GB, and then a hypoglycemic drug composed of ginsenoside monomer compounds could be found through *in vitro* hypoglycemic activity screening and *in vivo* hypoglycemic activity verification, which had more efficient hypoglycemic activity and could be used to replace the original total ginsenoside extract. It was conducive to drug quality control as well as modern development of hypoglycemic effect of GB.

Ferroptosis was a new programmed cell death mode, which different from apoptosis and autophagy. Its main mechanism was that under the action of divalent iron or lipoxygenase, unsaturated fatty acids on cell membrane produce lipid peroxidation, and then induced cell death (29). It was reported that pancreatic iron deposition will occur during the development of T2DM, which will lead to pancreatic β-cells dysfunction and death. The inhibition of pancreatic iron deposition and pancreatic β-cells ferroptosis had a beneficial regulatory effect on T2DM, indicating that ferroptosis was closely related to the occurrence and development of T2DM, and its internal relationship with T2DM was worthy of further study (30).

## Conclusions

In the present study, the total ginsenosides extract of GB was extracted by organic solvent extraction, and 5 extraction fractions of GB with different polarity were obtained. The insulin resistance model of HepG2 cells was established, and the hypoglycemic active fraction of five extracted fractions were screened *in vitro*. It was found that GBE-5 fraction had better hypoglycemic activity than other fractions, which was the hypoglycemic active fraction of GBE. The main components of GBE-5 fraction were found to be ginsenoside Rg_1_, Re, Rc, Rd, Rb_1_, Rb_2_, Rb_3_, and Rg_3_. The T2DM rat model induced by HFD/STZ was established, and the hypoglycemic activity of ginsenoside GBE-5 was evaluated. It was found that ginsenoside GBE-5 had significant hypoglycemic activity, the fasting blood glucose decreased from 13.44 ± 2.99 mmol·L^−1^ to 8.77 ± 4.91 mmol·L^−1^ (*p* < 0.05), and the blood glucose decreased significantly by 34.75%, the hypoglycemic effect was better than the total ginsenosides extract of GB (the hypoglycemic rate was 27.35 %). Univariate statistical analysis based on UHPLC–MS was used to screen the metabolites with different contents in the serum of rats after 30 days of intervention. Combined with the enrichment analysis of KEGG metabolic pathway, 18 differential metabolites that could be enriched into KEGG metabolic pathway were screened and identified in serum of T2DM *vs*. GBE-5 group. The metabolic pathways involved in regulation mainly include AA metabolism, LA metabolism, biosynthesis of unsaturated fatty acids and ferroptosis, which were potential targets for hypoglycemic activity of GBE-5 fraction of GB. In this study, the GBE-5 active fraction and its main hypoglycemic mechanism were studied, which will lay a foundation for the further development and utilization of *P. ginseng* berry as health functional food or dietary supplement.

## Data Availability Statement

The original contributions presented in the study are included in the article/supplementary material, further inquiries can be directed to the corresponding author/s.

## Ethics Statement

The animal study was reviewed and approved by the Institutional Animal Care and Use Committee (IACUC) of the Changchun University of Chinese Medicine with the permit code CPCCUCM IACUC 2019-009.

## Author Contributions

This study was designed by WW and HW. The sample collection, detection, and data curation were done by HW, YT, and AW. The data analysis and interpretation were exercised by WW, HW, and YL. The software, supervision, and validation were dealt by HW, HL, LJ, and BL. The writing of original draft was carried out by HW. Writing-review and editing and final approval of the published version were done by WW. All authors read and approved the final manuscript.

## Funding

This research work was supported by Jilin Scientific and Technological Development Program (No. 20200301049RQ) and National College Students Innovation and Entrepreneurship Training Program (202110199015).

## Conflict of Interest

The authors declare that the research was conducted in the absence of any commercial or financial relationships that could be construed as a potential conflict of interest.

## Publisher's Note

All claims expressed in this article are solely those of the authors and do not necessarily represent those of their affiliated organizations, or those of the publisher, the editors and the reviewers. Any product that may be evaluated in this article, or claim that may be made by its manufacturer, is not guaranteed or endorsed by the publisher.
